# Sarcoidosis-Associated Pulmonary Hypertension

**DOI:** 10.3390/medicina61020342

**Published:** 2025-02-14

**Authors:** Yoshitaka Morimatsu, Nobuhiro Tahara, Masaki Okamoto, Munehisa Bekki, Atsuko Tahara, Yoshiko Eto, Tadahiro Kugai, Yuki Koga, Shoko Maeda-Ogata, Akihiro Honda, Sachiyo Igata, Yoshiaki Zaizen, Shuichi Tanoue, Tomoaki Hoshino, Tatsuya Ishitake, Yoshihiro Fukumoto

**Affiliations:** 1 Department of Environmental Medicine, Kurume University School of Medicine, Kurume 830-0011, Japan; 2 Division of Cardiovascular Medicine, Department of Medicine, Kurume University School of Medicine, Kurume 830-0011, Japan; 3 Department of Respirology, NHO Kyushu Medical Center, Fukuoka 810-0065, Japan; 4 Division of Respirology, Neurology and Rheumatology, Department of Internal Medicine, Kurume University School of Medicine, Kurume 830-0011, Japan; 5 Department of Radiology, Kurume University School of Medicine, Kurume 830-0011, Japan

**Keywords:** sarcoidosis, sarcoidosis-associated pulmonary hypertension, epidemiology, pathogenesis, treatment

## Abstract

Sarcoidosis is a granulomatous disorder of unknown etiology characterized by multisystem non-caseating granulomas. Pulmonary hypertension (PH) is a well-known complication of sarcoidosis and is associated with increased morbidity and mortality. The actual epidemiology of sarcoidosis-associated PH (SAPH) remains unknown, and its pathogenesis has not been fully elucidated. SAPH is classified under the miscellaneous category (group 5 of the PH classification). The clinical presentation of SAPH is variable and not always proportional to the severity of sarcoidosis. Appropriate management for SAPH by an experienced physician is important; however, no treatment algorithm for SAPH has been established. Lung transplantation should be considered in refractory cases. Pulmonary arterial hypertension-specific vasodilators targeting the endothelin pathway, nitric oxide pathway, and prostacyclin pathway have improved the clinical functions and hemodynamics in some patients with SAPH.

## 1. Introduction

Sarcoidosis, which causes lesions in organs throughout the body, was reported to be associated with pulmonary hypertension (PH) in the 1980s [1]. Sarcoidosis-associated PH (SAPH) is an independent factor for a substantially increased risk of sarcoidosis mortality and a decline in functional capacity. Previously, SAPH was simply considered to be secondary to lung diseases; however, some patients with SAPH do not have lung fibrosis and lung parenchyma involvement [2,3,4,5]. Because its pathogenesis is multifactorial, understanding the etiology of SAPH is essential to selecting an appropriate treatment strategy. Treatment involves a combination of sarcoidosis-oriented therapy and PH-oriented therapy, but there are currently no specific treatment recommendations for SAPH. Therefore, treatment for SAPH should be considered on an individual basis.

## 2. Definition of SAPH

PH is a hemodynamic state of elevated blood pressure in the pulmonary artery comprising many conditions. Transthoracic echocardiography (TTE) is an initial screening tool for PH, but right heart catheterization (RHC) is necessary to confirm the diagnosis. Its diagnosis is determined via RHC with mean pulmonary arterial pressure (PAP) > 20 mmHg defined as resting PH. Also, exercise PH has been introduced with a new definition [4,5,6,7]. The hemodynamic categories of PH are presented in Table 1. PH is classified into five main PH groups based on the World Health Organization (WHO) clinical classification. These are categorized according to clinical conditions based on similar PH pathophysiological mechanisms, clinical features, hemodynamic characteristics, and treatments [4,5,6,7]. Currently, it is acknowledged that sarcoidosis may lead to PH via multifactorial mechanisms, resulting in various forms of PH [4,5,6,7]. In PH clinical classification, SAPH belongs to the WHO group 5 (PH with unclear and/or multifactorial mechanisms). However, SAPH contains several elements of groups 1 to 5, depending on the clinical symptoms present in each patient [4,5,6,7].

## 3. Epidemiology and Prognosis

The frequency of SAPH is reported to be 5.3–28.3% in stable sarcoidosis [1,8,9] and 70–80% in patients with lung transplantation indications or in advanced cases [10]. In one study, 47% of patients with sarcoidosis who had unexplained shortness of breath had PH [11], and PH was confirmed by RHC in 20 of 130 (15.4%) patients with persistent shortness of breath after immunosuppressive treatment. Additionally, the frequency of SAPH evaluated via TTE was 28.5%, 22.2%, 38.8%, 26.7%, and 69.7% for sarcoidosis stages 1 to 4, respectively, indicating a particularly high complication rate of PH in stage 4 [12]. A cross-sectional study of 313 patients with biopsy-proven sarcoidosis showed that 37 (11.8%) patients had PH on TTE with systolic PAP > 40 mmHg, and 9 of 12 patients with left ventricular (LV) dysfunction had PH confirmed on RHC [13].

A study of 130 patients with sarcoidosis and persistent dyspnea despite immunosuppressive therapy found that the hazard ratio for death in patients with PH was 10.39, as compared with patients who did not have PH [11]. When adjusted for age and lung function, SAPH has a 7-fold increased mortality risk compared with sarcoidosis without PH [12]. According to a report examining the prognosis of 60 patients with sarcoidosis but without PH and 50 patients with SAPH, the number of deaths was high in 3 (5.5%) patients with sarcoidosis but without PH and in 18 (36%) patients with SAPH. The average survival time was 4.2 years in the SAPH group, showing a poor prognosis [9]. An estimated PAP > 50 mmHg on TTE was associated with worse mortality, whereas mortality was unchanged in patients with an estimated PAP < 30 mmHg [14]. SAPH ultimately leads to excessive mortality [11,14,15,16].

## 4. Mechanism of Pulmonary Hypertension in Sarcoidosis

Inflammation of the noncaseating granulomas in sarcoidosis may lead to fibrosis and/or failure of the affected organs [17]. Both inflammation and fibrosis play a role in SPAH development. In stage 4 sarcoidosis, fibrotic lung tissue causes clinical PH derived from pulmonary arteriosclerosis [18]. However, SAPH cannot be explained by pulmonary fibrosis alone because PH is complicated, even in stage 1–2 sarcoidosis without pulmonary fibrosis. Further, there is no correlation between pulmonary ventilation function and PAP. SAPH may result from parenchymal lung diseases, the extrinsic compression of pulmonary vessels, pulmonary vasculopathy, LV dysfunction, or portal hypertension [4]. The pathophysiology of SAPH is complex and involves multiple potential mechanisms.

### 4.1. Pulmonary Arterial Hypertension and Pulmonary Venous Disease

In sarcoidosis autopsy cases, granulomatous involvement has been observed at all levels, from large elastic pulmonary arteries to venules [19]. The extent of granulomatous vascular involvement is related to that of parenchymal granuloma. Granulomatous inflammation is found in all blood vessels and all layers. The pathologic features of PAH, such as intimal fibrosis and plexiform lesions, are also seen in SAPH [19,20]. PAH might be derived from panangiopathy of the pulmonary arteries, thereby resulting in increased pulmonary vascular resistance (PVR). However, venous involvement has been found to be more prominent than arterial involvement [19], suggesting the presence of pulmonary veno-occlusive disease and pulmonary venous disease.

### 4.2. Pulmonary Hypertension Secondary to Left Heart Disease

Pulmonary arterial wedge pressure greater than 15 mmHg is necessary for the diagnosis of PH-related left heart disease (Table 1) [6]. The estimated co-occurrence of PH in patients with cardiac sarcoidosis ranges from 23% to 79%, owing to differences in diagnostic criteria between studies and heterogeneity of the study population [21]. Retrograde pressure transmission to pulmonary beds from the left heart is mainly caused by diastolic dysfunction. Additionally, increased retrograde pressure might introduce the release of vasoconstrictor molecules from pulmonary vessels, which can cause the “pre-capillary” component of PH in left heart disease. Furthermore, prolonged PH resulting from poorly controlled heart disease may lead to adaptive and eventually permanent vascular and cardiac remodeling [22]. Under this concept, the role of disturbed blood flow is an important trigger in the development of PH in left heart disease. It has been reported that catheter ablation for atrial fibrillation (AF) can cause iatrogenic PH [23]. However, in the propensity-matched analysis among 28 nonparoxysmal AF patients with PH undergoing a pulsed-field ablation (PFA)-based ablation procedure after >1 failed standard radiofrequency ablation (RFA), no worsening of mPAP was detected following PFA [24].

### 4.3. Pulmonary Hypertension Owing to Chronic Lung Disease

In a cohort study of 363 patients with sarcoidosis listed on lung transplantation registration, 73.8% had PH, and patients with PH required more supplemental oxygen than those without PH [10]. In multivariate analysis, supplemental oxygen remained an independent predictor of PH [10]. Parenchymal lung lesions are strongly associated with SAPH. However, pulmonary vasculature lesions coexist with parenchymal lung lesions and may independently contribute to SAPH. Patients with heart failure are at increased risk for sleep-disordered breathing, which may contribute to the development of PH in sarcoidosis [25]. Additionally, it has been demonstrated that patients with sarcoidosis have a higher rate of sleep-disordered breathing [26,27], resulting in an increased risk of developing PH secondary to nocturnal hypoxemia owing to obstructive sleep apnea [28].

### 4.4. Chronic Thromboembolic Pulmonary Hypertension (CTEPH)

CTEPH develops owing to pulmonary emboli that become organized fibrous scars, eventually leading to small vessel thrombosis and plexiform lesions [29,30]. Sarcoidosis confers an elevated risk of venous thromboembolism, which is thought to be related to active inflammation promoting a hypercoagulable state [31]. A cohort analysis elucidated a 3-fold increase in the hazard ratio of venous thromboembolism in patients with sarcoidosis [32]. Also, patients with sarcoidosis under age 65 years had a risk ratio of 2.0 for pulmonary embolism (95% confidence interval: 1.1–3.4) [33]. However, CTEPH occurs in up to 9% of patients at 2 years after an acute pulmonary embolism. Serum levels of inflammatory biomarkers, including C-reactive protein, interleukin (IL)-1, IL-2, IL-4, and IL-8, have been identified as risk factors for CTEPH [29]. However, it remains unclear why CTEPH develops in some patients [30].

### 4.5. Other Mechanisms of SAPH

In patients with marked thoracic lymphadenopathy and fibrous mediastinitis, structural strains of the major branches of the pulmonary circulation may cause physical impedance to pulmonary blood flow, resulting in pulmonary vascular stenosis and segmental PH [34,35]. Also, the possibility of portopulmonary hypertension should be considered in patients with sarcoidosis and liver involvement. However, portal hypertension develops in less than 1% of patients with sarcoidosis [36]. SAPH patients with radiological cystic-like lesions or cavities may require differentiation from pulmonary Langerhans histiocytosis or lymphangioleiomyomatosis-associated PH. The upper lobe-predominant distribution, the non-irregular cyst wall, and the presence of nodular shadows on HRCT in SAPH patients are useful in distinguishing these diseases [37]. The overall incidence of these etiologies appears to be quite rare; these should not be forgotten in a differential workup of SAPH.

## 5. Diagnosis of SAPH

**Figure 1:**
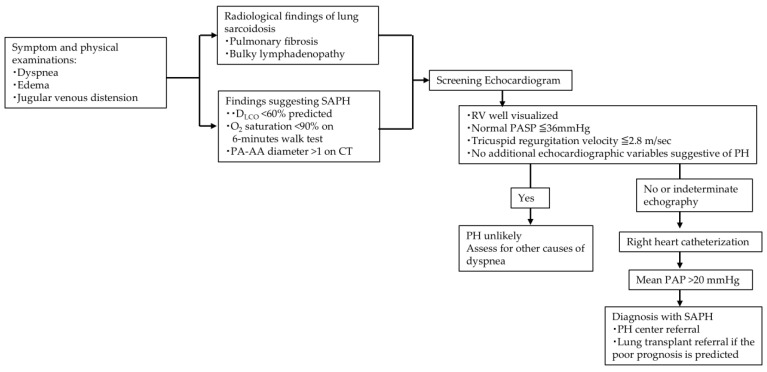
Screening algorithm of SAPH. CT, computed tomography; SAPH, sarcoidosis-associated pulmonary hypertension; PA, pulmonary artery; AA, ascending aorta; PASP, pulmonary artery systolic pressure; PAP, pulmonary arterial pressure; RV, right ventricle.

SAPH should be strongly suspected in patients with sarcoidosis who have worsening dyspnea or signs of right-sided heart failure. Dyspnea may appear refractory to immune suppression. Dyspnea and individual treatment strategies should be assessed in detail before selecting a treatment approach. A screening algorithm for SAPH is shown in Figure 1.

### 5.1. Medical History and Physical Examination

Symptoms of SAPH include exertional dyspnea, chest pain, palpitation, and syncope. Advanced SAPH shows increased pulmonary II sound, jugular vein distension, right ventricular heave, and peripheral edema. Ambulating hypoxemia is a key feature of PH [38].

### 5.2. Chest X-Ray and CT Scan

The presence of advanced lung disease on chest X-ray has been associated with SAPH [7,11]. However, PH developed in 40% of patients with sarcoidosis in the absence of radiographic features of advanced lung disease [12]. SAPH may exist in isolation of serious lung disease [39]. The diameter of the pulmonary artery relative to the bifurcation level of the aorta has been used to detect PH on computed tomography (CT) scans. In a sarcoidosis-specific study including more than half of patients with an advanced radiographic stage of pulmonary sarcoidosis (Scadding stage IV), the CT-measured pulmonary artery diameter corrected for body surface area was the best predictor of SAPH [40]. However, the reliability of chest CT parameters for predicting PH is limited. A formal PH workup should be performed for patients with suspected SAPH who have parenchymal lung disease [41].

### 5.3. Pulmonary Function and Exercise Test

Routine pulmonary function tests may provide clues to the presence of PH in patients with sarcoidosis. The diffusing capacity of the lung for carbon monoxide (D_LCO_), forced vital capacity, and 6 min walk distance (6MWD) is decreased in patients with SAPH [42]. In particular, D_LCO_ and desaturation during a 6 min walk test (6MWT) are the strongest predictors of SAPH, reflecting capillary desaturation from high pulmonary pressure and circulatory insufficiency [43]. In a study of 162 patients with sarcoidosis, a D_LCO_ < 60% and oxygen saturation < 90% during a 6MWT predicted PH with odds ratios of 7.3 and 12.1, respectively [42]. Both FVC and D_LCO_ can be decreased in sarcoidosis patients with lung fibrosis, whereas only D_LCO_ can be decreased in SAPH patients without lung disease. So, the discrepancy between FVC and D_LCO_ is useful for distinguishing between lung fibrosis and PH in sarcoidosis patients [42].

### 5.4. Echocardiography and Cardiac Catheter

**Figure 2:**
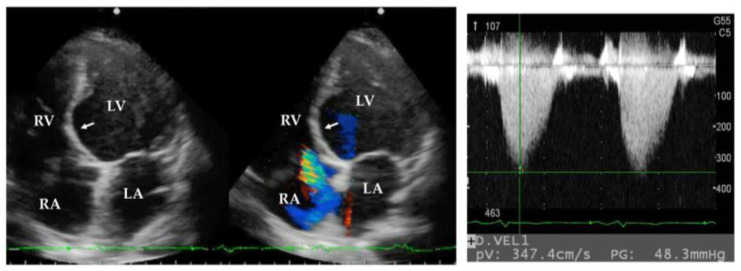
Transthoracic echocardiographic images of a patient with SAPH. Transthoracic echocardiography demonstrates a septal thinning (arrow) and regurgitant tricuspid jet, suggesting pulmonary hypertension in a patient with cardiac sarcoidosis. SAPH, sarcoidosis-associated pulmonary hypertension; RA, right atrium; RV, right ventricle; LA, left atrium; LV, left ventricle.

TTE is a non-invasive method to screen for PH from all causes. TTE is particularly suitable for patients with sarcoidosis because cardiac evaluation can be performed at the same time [44,45]. An estimated PAP can be assessed by regurgitant tricuspid jet on TTE (Figure 2), but tricuspid regurgitant is not detected in all patients. Additionally, the accuracy of the estimated PAP is reduced in patients with fibrotic lung disease. Therefore, TTE should not be used as a definitive PH diagnostic tool. RHC is the gold standard method for the diagnosis of PH. In a cross-sectional study of patients with idiopathic pulmonary fibrosis using TTE and RHC data, TTE could accurately estimate mean PAP in approximately 40% of patients [46].

### 5.5. 18F-Fluorodeoxyglucose-Positron Emission Tomography (FDG-PET)

**Figure 3:**
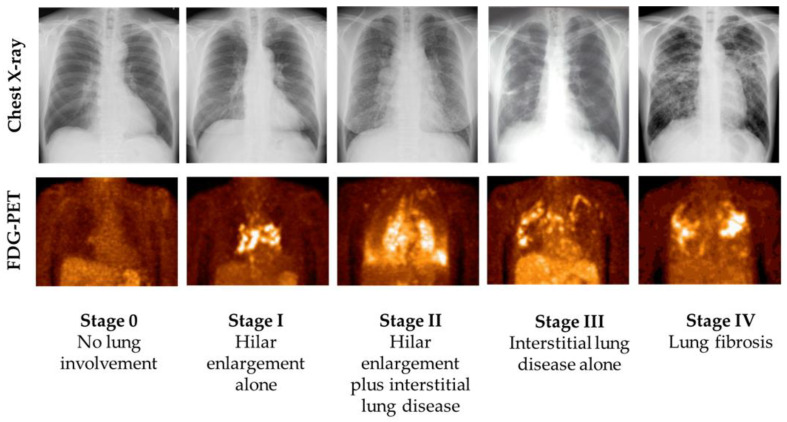
Chest X-ray and FDG-PET images according to the Scadding stage. 18F-Fluorodeoxyglucose-positron emission tomography (FDG-PET) showing FDG uptake in inflammatory lesions of pulmonary sarcoidosis.

**Figure 4:**
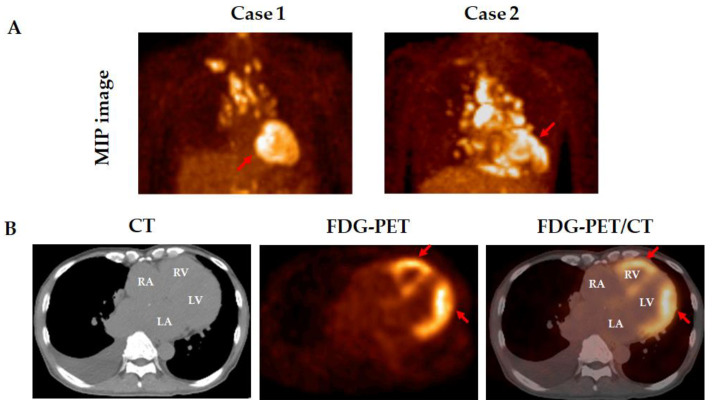
Representative sarcoidosis cases with FDG uptake in the heart. 18F-Fluorodeoxyglucose-positron emission tomography (FDG-PET) showing FDG uptake in the left ventricle (**A**). FDG uptake is shown in the right and left ventricle (**B**). FDG uptake lesion were pointed by red arrow. CT, computed tomography; MIP, maximum intensity projection; RA, right atrium; RV, right ventricle; LA, left atrium; LV, left ventricle.

Active inflammation in granulomatous lesions is seen in most patients with fibrotic pulmonary sarcoidosis [47,48]. FDG-PET has become the mainstay noninvasive imaging modality for the assessment of inflammatory activity in patients with sarcoidosis (Figure 3) [49]. Up to 85% of patients with stage 4 pulmonary sarcoidosis show active inflammation in lung parenchyma on FDG-PET images [50,51,52]. Also, preparation prior to the FDG-PET scan, including high-fat, low-carbohydrate diet modification and a more than 18 h fast, is a promising method for identifying inflammatory myocardial lesions in patients with sarcoidosis who have cardiac involvement (Figure 4A) [53]. In cardiac sarcoidosis, FDG uptake is seen not only in the LV but also in the right ventricle (RV) (Figure 4B). Besides ongoing active myocardial inflammation, RV overload derived from PH may also contribute to FDG uptake in the RV. Notably, FDG uptake in the RV has been associated with adverse events, including lethal arrhythmia and all-cause death in patients with cardiac sarcoidosis [54]. In that study, the mean PAP in patients with adverse events was significantly higher than in those without adverse events (24.0 ± 7.6 mmHg vs. 15.9 ± 3.1 mmHg, *p* < 0.001) [54]. Among 30 patients with active cardiac sarcoidosis who underwent RHC, 8 (26.7%) patients had SAPH, and 4 (13.3%) patients presented a precapillary PH phenotype [54]. Cardiac magnetic resonance imaging (MRI) and FDG-PET have assumed a valuable role in the diagnosis of patients with cardiac sarcoidosis [17].

## 6. Treatments

The pathophysiology of SAPH holds relevance for treatment. Current recommendations are to treat the underlying cause of the disease. The treatment strategy for patients with SAPH is to select sarcoidosis-oriented treatment, treatments for the cause of secondary PH, and PAH-specific therapy based on the pathological conditions of the individual patient. Several small studies conducted at expert centers have shown that pulmonary vasodilators are effective for SAPH, indicating a pre-capillary PH phenotype. Table 2 summarizes the main findings. Notably, pulmonary vasodilators may worsen the clinical condition of patients with SAPH who have pulmonary veno-occlusive conditions [55]. Medications such as diuretics, afterload reduction agents, and beta blockers are the mainstays of treatment for WHO group II PH. However, empiric therapy should not be performed because of the high risk of developing heart failure and pulmonary edema.

### 6.1. Corticosteroids

The mainstay of treatment for patients with sarcoidosis is systemic corticosteroids if disease activity is high. Corticosteroids impede the formation of granulomas and are effective against most active clinical manifestations of sarcoidosis. However, the efficacy of corticosteroids for SAPH has not been fully clarified. In a study including a small number of patients with SAPH, 10 patients (stage 0: *n* = 1, stage II: *n* = 4, and stage IV: *n* = 5) among 22 patients received high-dose oral prednisolone, which resulted in a >20% reduction in systolic PAP, suggesting that corticosteroid treatment might resolve PH-related granulomatous inflammation [56]. However, other studies have not shown significant hemodynamic improvements in stage IV sarcoidosis treated with corticosteroids [57,67,68]. Additionally, long-term use of corticosteroids can cause serious adverse effects [69,70]. Moreover, fibrocystic sarcoidosis does not respond to corticosteroids [71,72,73]. Corticosteroid therapy may be suitable for patients with SAPH who have pulmonary artery compression owing to active granulomatous inflammation or lymphadenopathy.

### 6.2. Anti-Inflammatory and Immunomodulatory Therapies

In a cohort study of SAPH, only 4 of 11 (36.4%) patients treated with immunosuppressant alone experienced improvement in pulmonary hemodynamics [58]. Methotrexate or azathioprine could have a potential therapeutic role as second-line treatment for patients with SAPH refractory to corticosteroids [74,75,76]. Also, anti-tumor necrosis factor monoclonal antibodies or rituximab might serve as a third-line treatment for SAPH [77,78]. However, there are no clinical data indicating the therapeutic benefits of these agents for patients with SAPH [77,78].

### 6.3. Endothelin Receptor Antagonists

Endothelin is a potent endogenous vasoactive molecule implicated in the pathophysiology of PAH via its ability to increase pulmonary vascular tone and exert long-term effects of pulmonary vascular remodeling [67]. Bosentan, ambrisentan, and macitentan are endothelin receptor antagonists currently available for clinical use. Endothelin receptor antagonists may block the activity of endothelin on pulmonary vascular smooth muscle in patients with SAPH [79].

A 16-week, double-blind, placebo-controlled, randomized clinical trial examined the efficacy of bosentan in patients with SAPH [16]. Of 39 patients, 4 (10.3%) dropped out. However, liver toxicity, a common side effect of bosentan, was not found during the study period. The trial demonstrated a significant reduction in mean PAP (11.1%) and PVR (25.4%) in 23 patients assigned to bosentan but no significant change in 12 patients assigned to placebo. No significant change in the 6MWD was noted in either group. A retrospective cohort study showed improvements in mean PAP, PVR, New York Heart Association (NYHA) functional class, and 6MWD in 22 patients with SAPH, 12 of whom received bosentan [59]. The efficacy of bosentan was particularly pronounced in patients with SAPH and a low radiographic stage of pulmonary sarcoidosis (Scadding stage; Figure 2) and high forced vital capacity (>50% of predicted values) [50,59]. Therefore, the presence of severe pulmonary fibrosis in SAPH appears to be associated with failure to respond to treatment.

A 24-week prospective, open-label, proof-of-concept trial of ambrisentan in 21 patients with SAPH demonstrated no significant changes in 6MWD, Borg dyspnea scale, brain natriuretic peptide, D_LCO_, and quality of life [51]. The study had a high dropout rate; 11 of the 21 (52.4%) patients did not complete the 24-week study [63]. The remaining 10 patients who completed the study showed improvement in WHO functional class and quality of life, although the differences were not statistically significant [63].

A retrospective study showed that macitentan treatment for longer than 12 months was well tolerated in five of six patients with SAPH [64]. In the study, four patients showed improvement in their NYHA functional class, and three patients had improved exercise capacity after 12 months of macitentan therapy [64].

### 6.4. Phosphodiesterase 5 Inhibitors and Soluble Guanylyl Cyclase Activator

Phosphodiesterase inhibitors suppress the degradation of intracellular cyclic guanylyl monophosphate in vascular smooth muscle, leading to increased nitric oxide generation and subsequent smooth muscle relaxation. Sildenafil and tadalafil, both members of this class, are available for clinical use. Riociguat, a soluble guanylyl cyclase activator, also serves to increase intracellular cyclic guanylyl monophosphate and is approved in group I and group IV PH [80]. However, it has not been assessed in patients with SAPH.

In a single-center retrospective study of 12 patients with end-stage pulmonary sarcoidosis referred for lung transplantation, sildenafil treatment for 4 months (range 1 to 12 months) showed significant reductions in mean PAP and PVR and a significant increase in cardiac output [65]. However, a significant change in 6MWT has also been observed [65].

In a 24-week open-label trial of tadalafil for SAPH at two academic medical centers, 12 patients with SAPH were enrolled over a period of 29 months. Of these, five (41.6%) patients dropped out of the study. Although there were no significant improvements in 6MWD, quality of life, or Borg dyspnea scale, three of six patients with Scadding stage III experienced a decrease in 6MWD [66].

### 6.5. Prostacyclins

Prostacyclin is a potent pulmonary vasodilator that inhibits platelet aggregation, causing relaxation of smooth muscle and vasodilation of the pulmonary arteries. Epoprostenol, treprostinil, and iloprost are prostacyclins available for PAH treatment. Formulations for prostacyclin analogs, including continuous intravenous infusion (epoprostenol and treprostinil), subcutaneous (treprostinil), oral (treprostinil), and inhaled (iloprost), have been available for patients with PAH. More recently, selexipag, a non-prostanoid prostacyclin receptor agonist, was shown to reduce hospitalization and disease progression [81]; however, it has not been studied in patients with SAPH.

Epoprostenol is the first medication approved for the treatment of PAH and has shown improvement in the risk of mortality [82]. In a retrospective cohort study, seven patients with SAPH underwent treatment with intravenous epoprostenol; half of these patients had stage IV radiographic disease with moderate to severe SAPH and NYHA function class III or IV symptoms [62]. Six of the seven patients had a significant hemodynamic response with a >25% reduction in PVR; five patients continuing intravenous epoprostenol for a mean follow-up of 29 months showed clinical improvement, with a decrease of 1–2 levels in the NYHA functional class. A prospective, observational study reported a significant response to intravenous epoprostenol with a decrease of ≥20% in PVR and an increase of 25% in cardiac output among patients with moderate-to-severe SAPH [60]. In a retrospective study including 26 patients with severe SAPH, intravenous or subcutaneous prostacyclin therapy showed significant hemodynamic and clinical improvement [13].

In an open-label, single-arm clinical trial, 15 of 22 (68.2%) patients with SAPH completed 16 weeks of inhaled iloprost therapy and showed a decrement of >20% in PVR. A ≥5 mmHg reduction in mean PAP and improved quality of life were found in five patients. Three patients had an increase in 6MWD by at least 30 m, and one had a decrease in PVR [61]. Monotherapy with inhaled iloprost could improve the pulmonary hemodynamics and quality of life in patients with SAPH [61].

### 6.6. Nitric Oxide

Inhaled nitric oxide was used for both acute vasoreactivity testing and for long-term treatment in eight patients with moderate to severe SAPH [60]. Seven of these eight patients had a ≥20% reduction in PVR and mean PAP at rest and mean PAP during an inhaled challenge with nitric oxide. One patient had improved 6MWD in combination with epoprostenol, and three patients had improvement in NYHA class.

The results of studies investigating the treatment of SAPH with pulmonary vasodilators have demonstrated improvements in patients’ hemodynamics, exercise capacity, and functional status. Pulmonary vasodilators have not been associated with harm in patients with SAPH selected by expert physicians. However, no studies were able to clearly demonstrate a benefit in terms of mortality. Many studies are limited by a small number of participants and a high dropout rate. Prospective, controlled trials of pulmonary vasodilators are warranted to verify apparent benefits in patients with SAPH.

In the clinical practice of PH, studies of precision medicine using biomarkers have significance. Mutations of the bone morphogenetic protein receptor type II (BMPR2) gene (encoding bone morphogenetic protein receptor type 2) have been identified as the most common cause of heritable PAH. The meta-analysis of individual participant data of 1550 patients with idiopathic, heritable, and anorexigen-associated PAH from 8 cohorts showed that patients with BMPR2 mutations present at a younger age with more severe disease are at increased risk of death or transplantation, compared with those without BMPR2 mutations [83]. In the prospective cohort study (DELPHI-2), asymptomatic BMPR2 mutation carriers yielded a PAH incidence of 2.3% per year (0.99% per year in males and 3.5% per year in females), and all PAH cases remained at a low-risk status with oral therapy at last follow-up [84]. Disease-causing genes within the transforming growth factor (TGF) superfamily, to which BMPR2 belongs, such as ACVRL1, ENG, and SMAD9, have been identified one after another. Furthermore, with the spread of next-generation sequencers, the number of causative genes and candidate causative genes has increased [85]. These genetic studies have the potential to become the basis for the development of precision medicine for PH, which may also be applied to the treatment of SAPH.

## 7. Conclusions

Clinical features of SAPH include a reduced D_LCO_ of <60%, oxygen desaturation < 90% on 6MWT, as well as refractory dyspnea after immune suppression. Because sarcoidosis is a systemic disease, the pathophysiology of SAPH is complex. The treatment strategy should be selected and the combinations determined according to individual pathological conditions. In recent years, a combination of PAH-specific agents has demonstrated beneficial effects in SAPH. It is important to always keep in mind the existence of SAPH in clinical settings. Early detection and early treatment may improve clinical outcomes of SAPH.

## Figures and Tables

**Table 1:** Hemodynamic categories of pulmonary hypertension.

PH Category	Definition
Pre-capillary PH	mPAP > 20 mmHg, PAWP ≤ 15 mmHg, PVR > 2 WU
Isolated post-capillary PH	mPAP > 20 mmHg, PAWP > 15 mmHg, PVR ≤ 2 WU
Combined pre- and post-capillary PH	mPAP > 20 mmHg, PAWP > 15 mmHg, PVR > 2 WU
Unclassified PH	mPAP > 20 mmHg, PAWP ≤ 15 mmHg, PVR ≤ 2 WU
Exercise PH	mPAP/CO slope between rest and exercise > 3 mmHg/L/min

Abbreviations: PH, pulmonary hypertension; mPAP, mean pulmonary arterial pressure; PAWP, pulmonary arterial wedge pressure; PVR, pulmonary vascular resistance; WU, Wood units; CO, cardiac output.

**Table 2:** Major findings of clinical studies in SAPH.

Treatment	Study Design	N	Primary Endpoint	Result	Ref.
Prednisolone	Retrospective /single arm	22	Survival	5-years survival is 59%	[56]
Prednisolone	Retrospective /single arm	24	1 year change in chest X-ray findings and pulmonary function	In half of patients, improvement of chest X-ray findings and pulmonary function	[57]
PAH-target therapy with or without immunosuppressive therapy	Retrospective /single arm	97	PVR, Exercise resistance, Survival	Significant decrease in PVR but not exercise resistance Survival at 1, 3, and 5 years was 93%, 74%, and 55%, respectively	[58]
PAH-target therapy	Retrospective /single arm	22	6MWD	Mean PAP, PVR, NYHA, and 6MWD were increased TFS at 1 and 3 years was 90% and 74%	[59]
Inhaled nitric oxide	Prospective /single arm	8	Mean PAP, PVR, 6MWD	Mean PAP was significantly improved, PVR was improved but not significantly, 6MWD was improved in all participants	[60]
Epoprostenol	Prospective /single arm	6	Mean PAP, PVR, 6MWD	4 of 6 patients showed improvement in hemodynamics	[60]
Inhaled iloprost	Prospective /single arm	22	Hemodynamics, 6MWD, SGRQ	PVR was improved in 6 patients, increase in 6MWD in 3, SGRQ was decreased in 7	[61]
Epoprostenol	Retrospective /single arm	8	Hemodynamics	Hemodynamics was significantly improved in 6 of 7 patients.	[62]
Prostacyclin	Retrospective /single arm	26	Hemodynamics, WHO-FC	CO, CI, PVR, BNP, and WHO-FC were significantly improved Eight and 5 patients survived at 5 and 8 years, respectively	[14]
Bosentan	double-blind, placebo-controlled trial	35	Mean PAP, PVR, 6MWD	Mean PAP and PVR were significantly reduced in treatment group but not placebo. 6MWD was not significantly changed in either group.	[16]
Ambrisentan	Prospective, open-label /single arm	21	Borg scale, WHO-FC, BNP, D_LCO_, Short Form-36, SGRQ	Only WHO-FC and SGRQ were improved but not significantly	[63]
Macitentan	Retrospective /single arm	6	WHO-FC, Exercise capacity	WHO-FC was improved in 4 patients, exercise capacity was improved in 3, 1 patient died	[64]
Sildenafil	Retrospective /single arm	24	Hemodynamics, 6MWD	MPAP, PVR, CO, CI was significantly improved, but 6MWD was not significantly changed.	[65]
Tadarafil	Retrospective /single arm	12	6MWD, Short Form-36, SGRQ, Borg scale	All endpoints were not significantly improved.	[66]

SAPH, sarcoidosis-associated pulmonary hypertension; PAH, pulmonary arterial hypertension; PVR, pulmonary vascular resistance; 6MWD, 6-min walk distance; PAP, pulmonary arterial pressure; SGRQ, St. George’s Respiratory Questionnaire; WHO-FC, World Health Organization functional classification; BNP, brain natriuretic peptide; D_LCO_, diffusing capacity of the lungs for carbon monoxide; CO, cardiac output; CI, cardiac index.

## Data Availability

Not applicable.

## Abbreviations

The following abbreviations are used in this manuscript:

PH	Pulmonary hypertension
SAPH	Sarcoidosis-associated PH
TTE	Transthoracic echocardiography
RHC	Right heart catheterization
PAP	Pulmonary arterial pressure
WHO	World Health Organization
mPAP	Mean pulmonary arterial pressure
PAWP	Pulmonary arterial wedge pressure
PVR	Pulmonary vascular resistance
WU	Wood units
CO	Cardiac output
LV	Left ventricular
CTEPH	Chronic thromboembolic pulmonary hypertension
IL	Interleukin
AA	Ascending aorta
PASP	Pulmonary artery systolic pressure
FVC	Forced vital capacity
RV	Right ventricle
CT	Computed tomography
DLCO	Diffusing capacity of the lung for carbon monoxide
6MWD	6-min walk distance
6MWT	6-min walk test
FDG-PET	18F-Fluorodeoxyglucose-positron emission tomography
PAH	Pulmonary arterial hypertension
SGRQ	St. George’s Respiratory Questionnaire
WHO-FC	World Health Organization functional classification
BNP	Brain natriuretic peptide
CI	Cardiac index
NYHA	New York Heart Association
